# Retraction: Analysis of the factors influencing the college students’ employment willingness under the strategy of “strengthening the provincial capital”

**DOI:** 10.1371/journal.pone.0309771

**Published:** 2024-08-28

**Authors:** The PLOS ONE Editors

After this article [1] was published, concerns were identified about the integrity of the peer review process.

A member of the *PLOS ONE* Editorial Board who was not involved in the article’s pre-publication peer review reviewed the article and noted concerns including inadequate and inaccurate referencing; insufficient reporting of the methodology that prevents reproducibility and interpretation; discrepancies in the study design and results; errors in formulas; and unsupported conclusions. The *PLOS ONE* Editors concluded that the article does not meet the journal’s criteria for publication.

In light of the above concerns, the *PLOS ONE* Editors retract this article. We regret that the issues were not identified prior to the article’s publication.

The authors did not agree with the retraction.
